# The intrinsic variance of beauty judgment

**DOI:** 10.3758/s13414-023-02672-x

**Published:** 2023-03-14

**Authors:** Maria Pombo, Aenne A. Brielmann, Denis G. Pelli

**Affiliations:** 1 Department of Psychology, New York University, New York, NY 10003 USA; 2 Department of Computational Neuroscience, Max-Planck Institute for Biological Cybernetics, Tübingen, Germany; 3 Center for Neural Science, New York University, New York, NY 10003 USA

**Keywords:** Recall memory, Sequential dependence, Aesthetics, Repeated measures, Intrinsic variability, Subjective beauty judgments

## Abstract

Recall memory and sequential dependence threaten the independence of successive beauty ratings. Such independence is usually assumed when using repeated measures to estimate the intrinsic variance of a rating. We call “intrinsic” the variance of all possible responses that the participant could give on a trial. Variance arises within and across participants. In attributing the measured variance to sources, the first step is to assess how much is intrinsic. In seven experiments, we measure how much of the variability across beauty ratings can be attributed to recall memory and sequential dependence. With a set size of one, memory is a problem and contributes half the measured variance. However, we showed that for both beauty and ellipticity, with set size of nine or more, recall memory causes a mere 10% increase in the variance of repeated ratings. Moreover, we showed that as long as the stimuli are diverse (i.e., represent different object categories), sequential dependence does not affect the variance of beauty ratings. Lastly, the variance of beauty ratings increases in proportion to the 0.15 power of stimulus set size. We show that the beauty rating of a stimulus in a diverse set is affected by the stimulus set size and not the value of other stimuli. Overall, we conclude that the variance of repeated ratings is a good way to estimate the intrinsic variance of a beauty rating of a stimulus in a diverse set.

## Introduction

Sample means are the bread and butter of perception research and modeling (Loftus, 1996). Sometimes, psychologists focus on a given behavior, its individual differences across participants, and some factors that may explain those differences (e.g., Dijkstra & Barelds, 2009; Dowker, 2019; Kerkhof, 1985). Less often, psychologists consider the intrinsic variability of a rating. Presumably, the participant’s given answer is a random sample from a distribution of possible answers. What is the variance of that underlying distribution? Attempts to measure this variance typically assume that the distribution is “stationary,” not changing over the experimental session, and that the measured values are independent of the order of the measurements (Fiske & Rice, 1955; Hultsch et al., 2008).

With the ultimate goal of understanding how beauty ratings vary within and across individuals, we here focus on the intrinsic variability (i.e., variance) of a beauty rating. Note that what we call “intrinsic variability” is also called within-individual variability or intraindividual variability (Hershberger & Moskowitz, 2013). The majority of research done in empirical aesthetics focuses on comparing means of beauty ratings (Corradi et al., 2020), but some researchers have highlighted individual differences in beauty judgment (e.g., Axelsson, 2007; Chen et al., 2022; Isik & Vessel, 2021; Leder et al., 2019). For example, previous studies indicate that idiosyncratic aesthetic taste contributes three times as much variance as universal taste (mean beauty ratings) to beauty ratings (Brielmann & Pelli, 2019; Leder et al., 2016; Vessel et al., 2018). In fact, several studies have aimed to partition the variance between shared and individual aesthetic ratings using various methods, including various correlation methods, variance component analyses, and beholder indices (e.g., Leder et al., 2016; Martinez et al., 2020; Vessel et al., 2018; Wallisch & Alden Whritner, 2017). Even though some of these methods rely on repeated measures, little is known about the intrinsic variability of a beauty rating.

The usual way to measure variance, in empirical aesthetics or elsewhere, is through repeated measures. One computes the variance across the participant’s responses to repeated presentations of the same stimulus, typically separated by responses to other stimuli. However, using the variance of repeated measures to estimate the intrinsic variance of a beauty rating is challenged by two phenomena that may wreck the assumed independence: memory and sequential dependence. Importantly, if repeated measures of beauty judgment are biased by memory or sequential dependence, measures of intrinsic variance of beauty judgment relying on repeated measures (e.g., beholder indices) are biased too, questioning the validity of the conclusions obtained from these measures.

Below, we review the memory and sequential dependence literature, especially in relation to beauty judgment.

### Memory

Memory can compromise the independence of repeated measures. Since individuals aim for consistency in their responses, stimulus or response memory could result in underestimating the intrinsic variance (Cialdini et al., 1995; Tourangeau, 2020). Seeing an image again may result in a feeling of familiarity (i.e., recognition). However, what we care about here is not whether an image seems familiar but whether a participant can recall its rating. Thus, we are interested in whether a new rating is influenced by a previous rating and not whether the image seems familiar. Could we estimate and discount the effects of memory on the estimated variance of judgment?

Psychologists distinguish between recognition and recall memory (Jacoby et al., 1993; Kopelman et al., 2007; Manns et al., 2003). *Recognition memory* is assessed by the ability to report whether a stimulus is new or old. *Recall memory* is assessed by the ability to report details of an old stimulus. Here, we are concerned with rating recall. Dependent measures of recall memory can be assessed through free recall or cued recall paradigms (Cleary, 2018). Free-recall memory is typically assessed by showing participants a set of elements and subsequently asking them to remember as many of them as possible. Cued-recall memory is typically assessed with a training task, in which participants observe a series of paired cues, and a test task, in which participants only see one of the cues in each pair and are asked to recall its corresponding cue. Though original paired-association learning paradigms used two-word pairings, others have explored multimodal pairings, such as pairing faces with regular nouns (Aue et al., 2017).

To date, little is known about how recall memory affects repeated measures. Schwarz et al. (2020), inspired by a previous study done by van Meurs and Saris (1995), aimed to calculate the effect of recall memory in a single repeated rating. In their study, participants were asked to answer a target question on a Likert scale. After answering a series of additional questions, participants got the target question again and were asked whether they remembered their answer. Participants who claimed to remember their answer were asked to reproduce that answer. Otherwise, they were asked to provide their best guess of that answer. They estimated that 17% of participants correctly reproduced their answers from memory. They did so by subtracting the base rate (proportion of participants who correctly reproduced their original answer despite claiming to not remember it) from the proportion of participants who remembered and reproduced their original answer. Though their study is a good starting point for understanding the effect of memory on repeated measures, it has some limitations. On one hand, their calculation of recall memory does not permit estimating the effect of recall memory on the variability of the repeated measures. On the other hand, their study only includes a single repeated measurement. The effect of recall memory on multiple repeated measures of judgment stills remains an open question.

The role of recall memory in repeated measures is of particular concern in empirical aesthetics research. A common practice in empirical aesthetics research is to ask participants to rate a set of images on various scales of aesthetic value (e.g., Brielmann & Pelli, 2019, 2020; Ishizu & Zeki, 2014; Marin et al., 2016; Vessel & Rubin, 2010). Humans are very good at remembering such images even if they have seen them only once (Standing, 1973). Hence, one can worry that recalling a previously rated image, which gives individuals a perceptual benefit, might influence subsequent ratings of the same image.

### Sequential dependence

In the presence of sequential dependence, order matters. The preceding section considered the possibility of sequential dependence due to recall memory, but it can arise in countless other ways as well. Sequential dependence could reflect effects of previous ratings or stimuli on the current rating. Two types of sequential dependence are commonly observed and have been extensively studied: assimilation and contrast effects. In assimilation effects, the percept of the current stimulus becomes more *like* other stimuli. In contrast effects, it becomes more *unlike* other stimuli. Josef Albers’ (1971) *Interactions of Color* provided many compelling demonstrations. In general, adding something nearby (e.g., a color) can make the original appear more similar to (*assimilation*) or more different from (*contrast*) the added item. The same terms are applied to corresponding response effects of a preceding stimulus (Ward & Lockhead, 1970).

One way to measure the influence of sequential dependence on the variability of ratings, for a given set of stimuli, is to compare the variance of the difference in repeated ratings between two blocks using the same sequence of stimuli versus that between two blocks using different sequences. In the presence of sequential dependence, the variance should be greater when the order differs.

Previous research has examined sequential dependence in aesthetic judgment. Huang et al. (2018) conducted five experiments through which they assessed the influence of stimulus modality and response type on contrast and assimilation effects. An assimilation effect means that, all else being equal, the current rating is more similar to the previous *rating*. A contrast effect means that, all else being equal, the current rating is biased away from the to-be-rated perceptual properties of the previous *stimulus*. They found that contrast and assimilation effects happen simultaneously but have different sources: assimilation effects stem from anchoring by the previous judgment and contrast effects stem from perceptual adaptation.

Their findings are consistent with other accounts of aesthetic ratings for faces assimilating to the previous response (Kondo et al., 2012; Taubert et al., 2016). The relationship between contrast effects and perceptual adaptation, at least in the context of facial attractiveness, have been contested (Kramer & Pustelnik, 2021; Xia et al., 2016). However, other studies looking at context effects on beauty ratings of photos suggest that contrast effects in beauty ratings persist regardless of the task instructions, the extent to which participants were warned of the context effects, and the insinuated similarity (e.g., participants were told the photos were all from the same photographer) between the contextual stimulus and the target stimulus (Tousignant & Bodner, 2014). Nevertheless, little is known about the effect of sequential dependence on the intrinsic variance of beauty judgment.

### Current study

The current study assesses the effects of memory and sequential dependence on the measured variability of beauty judgment. Ultimately, this allows us to use variance of repeated measures as an estimator of the intrinsic variability of a beauty rating, and other subjective ratings. The paper has three sections: memory discounting (Experiments 1 and 2), sequential dependence (3–6), and set-size effects (7).

Experiment 1 aims to discount the effect of memory on the variance of repeated measures of beauty judgment. Experiment 2 aims to replicate Experiment 1 with ellipticity ratings. Asking participants to rate the ellipticity of ellipses varying in aspect ratios allowed us to compare the results of Experiment 1 to a perceptual task with an objective truth. Doing so, we can test whether a memory-induced bias is exclusive to beauty rating. Experiment 3 measures the effect of sequential dependence on the intrinsic variability of beauty judgment. Previous literature suggests a stimulus similarity as a possible modulator of sequential dependency, so in Experiments 4, 5 and 6 we explore how that result generalizes to rating ellipticity and to rating beauty when the images are all similar to one another (sunsets and photoshoot images). Lastly, to ensure that our results are not exclusive to an arbitrary stimulus set size, Experiment 7 explores memory and sequential dependence effects as a function of stimulus set size. Overall, we assess the validity of repeated measures in different contexts, examining their appropriateness to estimate intrinsic variance of beauty judgment.

By assuming that similarity refers to the number of variable parameter dimensions between images, the stimuli in Experiments 3–6 provide us with a somewhat continuous measure of similarity. The Open Affective Standardized Image Set (OASIS) images, which vary along an infinite number of dimensions, are the most dissimilar, and the ellipses, which vary only along three dimensions, are the most similar. We consider the sunsets to be less similar than the photoshoot images since the photoshoot images are all of the same subject, conceivably maintaining the colors and shapes present in all stimuli constant.

## Experiment 1: Discounting the effect of recall memory on the variance of repeated beauty judgment

### Methods

#### Rationale

In order to assess how reliably participants remember ratings of images, we named the images and used the name to cue a remembered rating. By asking participants to provide a rating from just a name, we are triggering a memory of an image’s rating without presenting the image. This allows us to estimate how well the rating is remembered. Assuming that the name is tightly linked to the image, the measured variance estimates the variance of the remembered rating. A memory check validates the assumption.

#### Participants

We recruited 51 participants through Prolific Academic (https://prolific.co/) to take part in our experiment. Twenty-five of them identified themselves as female and 26 as male. Their ages ranged from 18 to 75 years (*M* = 33.06, *SD* = 13.71). All participants were U.S. nationals, spoke English as their first language, and indicated having normal or corrected-to-normal vision. All participants gave informed consent in accordance with the Declaration of Helsinki. This experiment was approved by the New York University Committee on Activities Involving Human Subjects (UCAIHS; IRB-FY2019-2456).

#### Stimuli and apparatus

We randomly selected 75 images from the 900 images of the OASIS database (Kurdi et al., 2017). According to the beauty ratings previously obtained by Brielmann and Pelli (2019), the mean beauty ratings (on a Likert scale of 1 = *not at all* to 7 = *very much*) for our subset of images ranged from 1.82 to 6.83 (*M* = 4.44, *SD* = 1.89). Every OASIS image is in one of four categories: animal, object, person, or scene. Of the 75 images we randomly selected, 12 were animals, 17 were objects, 25 were people, and 21 were scenes. The screen background was white. The name (14-pt Helvetica font, black) appeared near the top, the response slider (30 px high) was near the bottom, and the image was in between. Each element was horizontally centered. The image display size was 400 px by 400 px, which, on a 2,880-px by 1,800-px display, corresponds to about 5.3° by 5.3° of visual angle for an observer at a 50-cm distance from the screen. All images are the same size and have the same aspect ratio, so they all underwent the same transformation. This experiment was programmed as a survey on Qualtrics (https://www.qualtrics.com/). All participants were told that they must use a desktop or laptop computer to complete the study, not a smartphone or tablet, but we did not verify compliance.

#### Names

We assigned an arbitrary one-syllable name to each image. The association between images and names randomly varied between participants. Half of the names corresponded to names commonly given to females (e.g., Liv, Brooke, Rose), and half of them corresponded to names commonly given to males (e.g., John, Paul, Fred) (Manes, 2018).

#### Procedure

After giving consent and answering demographic questions about their age and gender, participants completed four blocks: initial, repeat, memory, and memory check. In the *initial* block, each participant saw each of the 75 images along with its accompanying name. The order of the images was randomized for each participant. Participants were asked to rate, on a scale from 1 (*not at all*) to 7 (*very much*) how much beauty they felt from looking at the image by shifting a slider appropriately. The initial position of the slider was leftmost, but even if participants wanted to rate the beauty of an image as 1, they still had to put the cursor on the slider and drag it. The slider did not include any tick marks and only included the end labels, both in numbers (1 or 7) and text (*not at all* or *very much*).

**Fig. 1:**
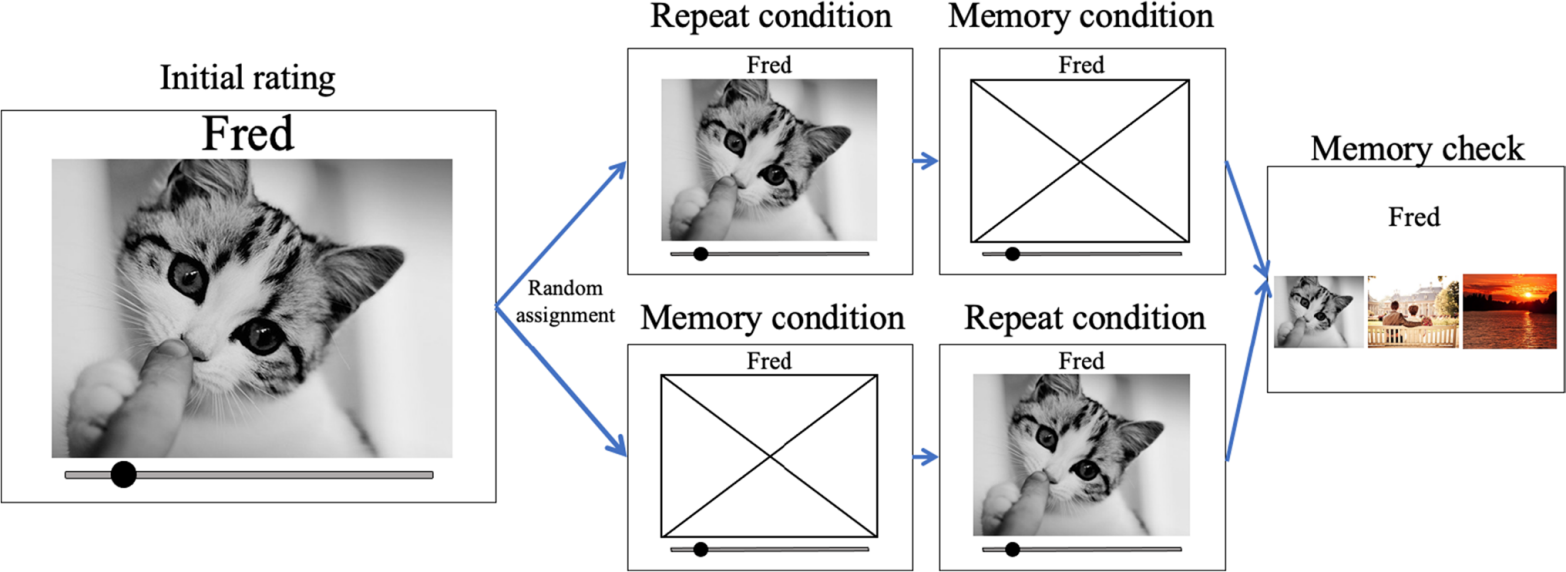
Graphical representation of the procedure of Experiment 1. Each box represents one block

Even though participants knew both ends of the scale (1 and 7), they were not told the saved numerical rating (rounded to two decimal places) corresponding to the slider location they set. The *repeat* block was the same as the initial one. In the *memory* block, participants saw each of the 75 names previously associated with one of the images. They were asked to remember the image associated with that name and rate, again on a scale from 1 (*not at all*) to 7 (*very much*), how much beauty they felt from that image. The order in which participants completed the repeat block and the memory block was counterbalanced. The last block was a *memory check*. Here, participants saw each of the 75 names previously associated with each image and were asked to select which of three images (all which were part of the 75-image set) was associated with that name. In the end, we had three beauty ratings (initial, repeat, and memory block) and a binary “remembered” versus “not-remembered” response for each image-name pair. Figure 1 shows a graphical representation of the procedure.

#### Analysis

All analyses were conducted using R (Version 4.0.5) in RStudio. Using the responses for which participants remembered the correct image–name association, we calculated the distribution of the difference between the beauty rating in the initial, repeat, and memory blocks. We then calculated the respective variances of the differences. For each distribution of differences we conducted a two-tailed one-sample *t* test to ensure that the mean difference was not significantly different from zero. This allowed us to test whether participant responses were higher or lower for a particular block. We also conducted an *F* test to assess whether the variances of the two distributions were equal. Doing so allowed us to estimate whether or not participant ratings were more variable when relying only on memory.

This is like a cue-combination paradigm, in which we treat the memory as a cue. In a wide range of perceptual judgments, observers combine cues optimally, following Bayes rule (e.g., Alais & Burr, 2019; Ernst & Banks, 2002; Oruç et al., 2003). For example, to understand someone’s speech, humans generally combine both the auditory cues of the person’s voice and the visual cues of their moving lips. To combine the visual and auditory cues optimally, the observer would combine the cues, weighing each cue by its reliability. Mathematically, reliability is one over variance $\left(\frac{1}{\sigma^2}\right)$. According to Bayes rule, the observer’s estimate will be a weighted sum of the cues, and the estimate’s reliability will be the sum of the cue reliabilities. In our model of the beauty task, the observer combines two cues: one from memory and one from their immediate perceptual experience. We estimate the memory reliability as the measured reliability when the participant can rely only on their memory of the image. In this model, the intrinsic variance of beauty rating is simply the variance of the immediate perception component.

The memory cue could reflect diverse traces, including a vivid recollection of the stimulus, their previous rating of the image, certain features of it (e.g., symmetry or color), or its poetic quality. We take the *memory cue* to be the beauty ratings in the memory block. The repeat block differs from the memory block solely by the presence of the stimulus, so we suppose that each repeat-block rating is the combination of contributions from the memory cue and the immediate-stimulus cue. Assuming optimal cue combination, and thus additivity of reliability, the total reliability $\frac{1}{\sigma_R^2}$ is the sum of the memory cue reliability $\frac{1}{\sigma_M^2}$ and the immediate perceptual reliability $\frac{1}{\sigma_I^2}$, which we can solve for the latter:

1 $$\frac{1}{\sigma_I^2}=\frac{1}{\sigma_R^2}-\frac{1}{\sigma_M^2}$$

where ${\sigma}_R^2$ and ${\sigma}_M^2$ are the variances of the combined and memory judgements respectively, and ${\sigma}_I^2$ is the variance of the immediate-perception judgment, an estimate of the intrinsic variance. To isolate the test–retest variance from the individual differences, we estimate the memory (or repeat) variance as the variance of the difference in beauty ratings between the initial and memory (or repeat) blocks.

Lastly, to determine the extent to which the beauty rating in the repeat and memory blocks predicts the initial beauty rating, we fit a linear mixed-effects model using the *lmer4* package in R (Bates et al., 2015). We included random intercepts for the image and participant. It is worth noting that even though intercept-only models may inflate Type I error, intercept-only models prevent us from getting singular models which are hard to interpret (Barr et al., 2013). The data and code for this and all subsequent experiments can be found here: https://osf.io/wecvp/.

### Results

We excluded the results of one participant who gave the same rating to every image in one of the blocks and therefore was not following the instructions (thus, *N* = 50). For our analyses, we considered the trials for which participants correctly identified which image (of three) was associated with each name. Of the memory-check trials, 59.5% (39.3% when corrected for guessing) were correct, where chance performance would be 33.3%.

**Fig. 2:**
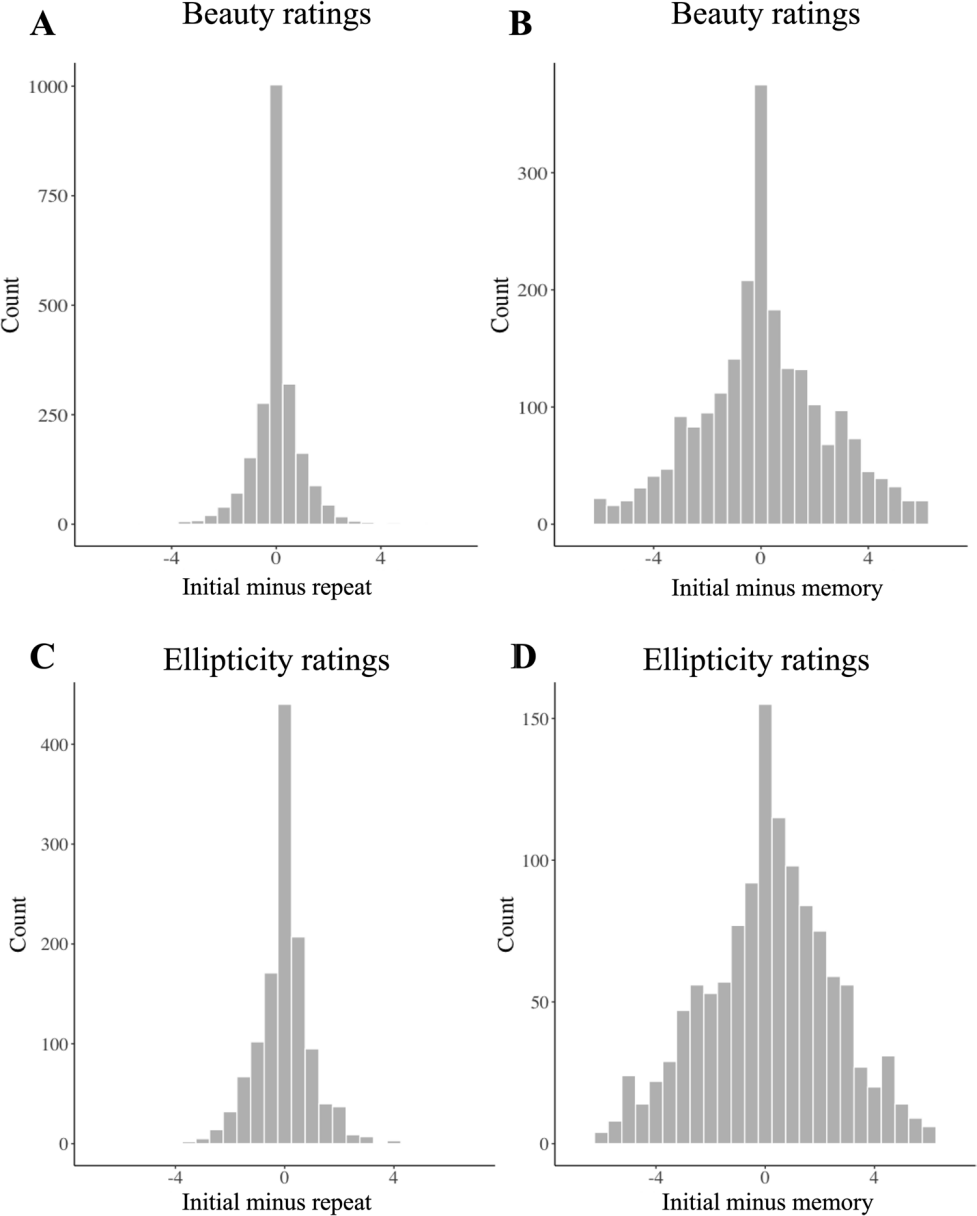
Histograms of the differences between the initial beauty rating and the beauty rating in the repeat block (**A**) and the memory block (**B**) and between the initial ellipticity rating and the ellipticity rating in the repeat (**C**) and memory (**D**) block

Figure 2A and B show the distributions of difference for the repeat block and memory block respectively. Table 1 displays the standard deviations of the repeat (*σ*_*R*_) and memory blocks. The ratio between these two standard deviations is significantly different from one, *F*(2226, 2226) = 0.1479, *p* < 0.001, 95% CI [0.136, 0.161].

**Table 1:** Standard deviation of difference for the repeat block, memory block, and immediate perception

Standard deviation of beauty ratings	
Standard deviation of repeat block (*σ*_*R*_)	0.91
Standard deviation of memory block	2.37
Corrected standard deviation of memory block (*σ*_*M*_)	2.23
The difference between the beauty ratings in the initial block and repeat blocks can be therefore thought of as the combination of the memory cue with an immediate-perception judgment. Standard deviation of immediate perception judgment (*σ*_*I*_) computed by Eq. 1.	1.00

The measured variance in the memory block is a mixture of ratings for recalled images and ratings for not-remembered images. Hence, our measure is an overestimation of the actual memory variance. We can correct for this using the variance of the memory block of the nonremembered images (incorrect trials in the memory block), in this case 2.64. Our correction for guessing indicates that 34% of the remembered trials are guesses, so a weighted average estimates the true *σ*_*M*_ to be 2.23.

The one-sample *t* tests indicate that, for each distribution, the mean difference is not significantly different from zero, *t*(2226) = 1.61, *p* = 0.11, *d* = 0.03, 95% CI [−0.007, 0.069] and *t*(2226) = 1.87, *p* = 0.062, *d* = 0.04, 95% CI [−0.005, 0.192] for the repeat and memory distributions, respectively. This indicates that, on average, participants’ beauty ratings were not biased in either direction for a particular block. Using the Bayesian optimal rule of cue combination described in Equation 1, we calculated the standard deviation of the immediate-perception judgment, *σ*_*I*_, to be 1.00 (see Table 1).

The results of our mixed-effects models indicate that while the initial beauty ratings predicted 81% of the variance of the beauty ratings in the repeat block, they only predicted 2% of the variance of the beauty ratings in the memory block. Tables 2 and 3 display details of the models for the repeat block and the memory block, respectively. Our results indicate a big disparity between the repeat block and the memory block. On average, for each point of increase in the initial beauty rating, the repeat-block beauty rating increases 0.9 points, and the memory-block beauty rating increases by just 0.13.

**Table 2:** Mixed-effects model for the repeat-block beauty rating

Random Effects (Intercepts)					
	Variance	*SD*			
Image	0.05	0.22			
Participant	0.06	0.24			
Fixed Effects					
	Estimate	*SE*	*df*	*t*	*p*
Intercept	0.32	0.06	164	5.10	<0.001
Initial Beauty Rating	0.90	0.01	727	80.83	<0.001

The model explains 81% of the variance in beauty ratings with an RMSE of 0.83

**Table 3:** Mixed-effects model for the memory-block beauty rating

Random Effects (Intercepts)					
	Variance	*SD*			
Image	0.04	0.20			
Participant	0.86	0.93			
Fixed Effects					
	Estimate	*SE*	*df*	*t*	*p*
Intercept	3.17	0.15	75	20.59	<0.001
Initial Beauty Rating	0.13	0.02	385	7.25	<0.001

The model explains 2% of the variance in beauty ratings with an RMSE of 1.51

With our design, half of the participants had an additional block between the memory condition and the memory check, allowing for an extra opportunity to encode the image-name pairings. To ensure that the block order did not interfere with the results, we estimated the ratio between the standard deviations (repeat vs. memory conditions) for both groups of participants. The results were very similar to each other and the combined results: *F*(578, 578) = 0.198, *p* < 0.001, 95% CI [0.168, 0.233] for those who completed the repeat condition first, and *F*(652, 652) = 0.1497, *p* < 0.001, 95% CI [0.128, 0.175] for those who completed the memory condition first.

### Discussion

After discounting the variance attributed to memory from the variance of repeated ratings (using Equation 1), our estimate of the variance of the immediate perception judgment is 0.09 larger (comparing *σ*_*R*_ and *σ*_*I*_ in Table 1). Therefore, our results indicate that the contribution of recall memory to repeated beauty ratings is small (we consider a 10% error in standard deviation negligible).

Though the stock images we used here are very memorable (Standing, 1973), it is conceivable that recall memory wrecks the independence of repeated measures in tasks other than beauty perception research. Thus, we redid the original experiment with a different task: We asked participants to rate the ellipticity of ellipses varying in aspect ratio. Rating ellipticity requires making a judgment about the state of the world rather than a judgment about how the world relates to oneself. By asking participants to rate ellipticity, we were able to assess the contribution of recall memory to repeated judgment in a context other than beauty and assess our paradigm by comparing ratings to an objective aspect ratio.

## Experiment 2: Discounting the effect of recall memory on the variance of ellipticity judgment

### Methods

#### Participants

We recruited 50 new participants for this experiment. Twenty-three of them identified themselves as female, 23 as male, one as other, and three preferred not to say. Their ages ranged from 21 to 62 years (*M* = 33.04, *SD* = 10.38). All participant selection and recruitment were the same as in Experiment 1.

#### Stimuli and apparatus

We generated 75 ellipses, which varied linearly in aspect ratio (the ratio of the width to the height of an ellipse) from 1:1 to 1:4 (i.e., *x* was uniformly spaced between 1 and 4 and the aspect ratio was 1/x). All the ellipses had the same area. On each trial, the major axis was randomly either horizontal or vertical. The color of each ellipse was specified by taking three random uniform samples (0 to 1) as an RGB color.

#### Procedure and analysis

There are two differences between Experiments 1 and 2. First, the task was different. Instead of asking participants how much beauty they felt from looking at the image, we told participants that each of the ellipses they saw used to be a perfect circle. We asked them to rate, on a scale from 1 (*not at all*) to 7 (*very much*) the extent to which the perfect circle had been squished. We refer to this rating as the ellipticity rating. Second, to ensure that the participants understood us and to anchor our scale, at the beginning of the study we showed participants that a minimal rating of 1 corresponds to a perfect circle, a maximal rating of 7 corresponds to a maximally squished ellipse, and a rating of 4 corresponds to an ellipse squished half as much. Before starting the experiment, participants completed three comprehension-check trials in which they were shown the exemplary figures and asked to rate them. Apart from the change in stimuli and task, and the additional instructions and practice trials, the procedure and analysis of Experiment 2 were the same as in Experiment 1.

### Results

**Fig. 3:**
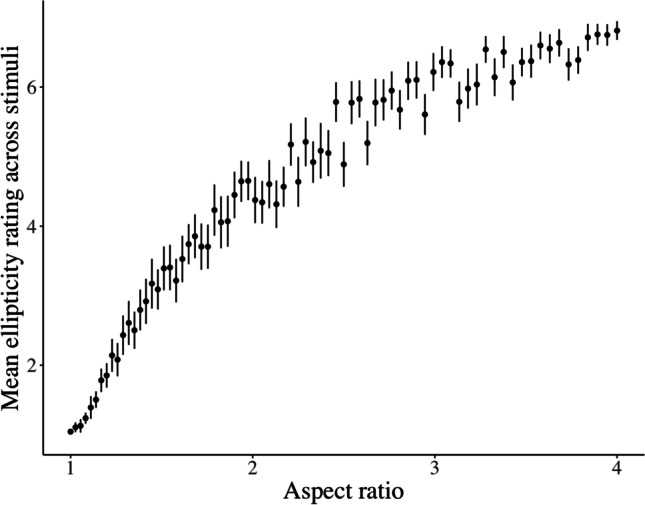
Mean ellipticity ratings vs. aspect ratio, across stimuli in Experiment 2. Confidence intervals represent ± two standard errors

We excluded the trials of the participants who failed to correctly answer the comprehension-check trials (eight participants, new *N* = 42). To test the efficacy of our ellipticity manipulation, we plotted the mean rated ellipticity against the true aspect ratio for each of our stimuli (Fig. 3). The plot is clearly nonlinear, showing a saturating monotonic increase.

We considered the trials for which participants correctly identified which image (of three) was associated with each name. This constitutes 39.1% of the data (8.7% when corrected for guessing). That means that most of the correct responses were just guesses and the participant remembered only 8.7% of the trials. Figure 2 panels C and D show the distributions of difference for the repeat block and memory block, respectively. Table 4 displays the standard deviations of the repeat (*σ*_*R*_) and memory blocks. The ratio between these two standard deviations is significantly different from one, *F*(1231, 1231) = 0.170, *p* < 0 .001, 95% CI [0.152 0.191].

**Table 4:** Standard deviation of difference for the repeat block, memory block, and immediate perception

Standard deviation of ellipticity ratings	
Standard deviation of repeat block (*σ*_*R*_)	0.97
Standard deviation of memory block	2.34
Corrected standard deviation of memory block (*σ*_*M*_)	2.23
Standard deviation of immediate perception judgment (*σ*_*I*_)	1.07

Again, the measured variance in the memory block is a mixture of ratings for recalled images and ratings for not-remembered images. Hence, our measure is an overestimation of the actual memory variance. We can correct for this using the variance of the memory block of the nonremembered images (incorrect trials in the memory block), in this case 2.37. Our correction for guessing indicates that 78% of the “remembered” trials are guesses, so a weighted average estimates the true *σ*_*M*_ to be 2.23.

One-sample *t* tests indicate that for the repeat block distribution (Fig. 2C) the mean difference is not significantly different from zero, *t*(1231) = −0.683, *p* = 0.49, *d* = 0.02, 95% CI [−0.073, 0.035]. However, for the memory block (Fig. 2D) the difference is significant, *t*(1231) = 2.34, *p* = 0.02, *d =* 0.07, 95% CI [0.025, 0.286]. This means that the mean ellipticity rating was slightly higher in the initial block than in the memory block (mean difference = 0.16). Using the Bayesian optimal rule of cue combination above (Eq. 1) we calculated the standard deviation of the immediate-perception judgment (*σ*_*I*_) to be 1.06.

The results of our mixed-effects models indicate that taking the initial ellipticity ratings as predictors of later ellipticity ratings, they predict 22% of the variance in the repeat block, but only 2% of the variance in the memory block. Note that in the memory block, including a random intercept term for the images, accounted for none of the variance in the ellipticity ratings, and thus we did not include it in the mixed-effect model reported. Tables 5 and 6 display model details for the repeat and memory blocks, respectively. Our results indicate that, on average, for each point increase in the initial ellipticity rating, the repeat-block ellipticity rating increases 0.36 points, and the memory-block ellipticity rating increases only 0.11.

**Table 5:** Mixed-effects model for the repeat-block ellipticity rating

Random Effects (Intercepts)					
	Variance	*SD*			
Image	1.04	1.02			
Participant	0.17	0.41			
Fixed Effects					
	Estimate	*SE*	*df*	*t*	*p*
Intercept	2.98	0.18	187.6	16.43	<0.001
Initial Ellipticity Rating	0.36	0.03	1062	13.61	<0.001

The model explains 22% of the variance in ellipticity ratings with an RMSE of 0.66

**Table 6:** Mixed-effects model for the ellipticity rating in the memory block

Random Effects (Intercepts)					
	Variance	*SD*			
Participant	0.11	0.33			
Fixed Effects					
	Estimate	*SE*	*df*	*t*	*p*
Intercept	3.85	0.13	340.3	30.317	<0.001
Initial Ellipticity Rating	0.11	0.02	1226	4.705	<0.001

The model explains 2% of the variance in ellipticity ratings with an RMSE of 1.55

### Discussion

Overall, our results indicate that discounting memory increases the variance of repeated ellipticity ratings by merely 0.1 (comparing *σ*_*R*_ and *σ*_*I*_ in Table 4), so the contribution of recall memory to repeated ellipticity ratings is minor. Together, Experiments 1 and 2 indicate that the contribution of recall memory to repeated judgments is below 10%, regardless of the objectivity of the measure.

The ellipses are generally similar in shape and hard to individuate. It is perhaps not surprising that they are hard to remember. Indeed, the guessing rate was much higher for ellipses than images, indicating that personal names are relatively ineffective as cues for ellipses. However, our variance estimates are corrected for guessing.

## Experiment 3: Sequential dependence in beauty ratings

As mentioned above, sequential dependence would wreck independence, and complicate the estimation of intrinsic variance from repeated measures of beauty judgment. We assess this effect by manipulating the order in which these judgments are made.

### Methods

#### Participants

Fifty new participants took part in the experiment. Twenty-nine identified themselves as female, 20 as male, and one as other. Their ages ranged from 18 to 67 years (*M* = 35.18, *SD* = 12.95). All participant selection and recruitment were the same as in the experiments above.

#### Stimuli and apparatus

The stimuli for this experiment were the exact same images as in Experiment 1. It was programmed and distributed in the same way as in Experiment 1.

#### Procedure

This experiment consisted of three blocks. In the initial block, participants rated the beauty of each of the 75 images on a scale from 1 (*not at all*) to 7 (*very much*) using a slider. In the same-order block, participants did the same thing and the images were presented in the same order as in the initial block. In the scrambled block, participants rated the beauty of the same images in the same way, except the order was scrambled. The order in which participants completed the same-order and the scrambled block was counterbalanced.

#### Analyses

We calculated the distribution of difference between the initial beauty ratings and the ratings in the same-order and the scrambled blocks. We also conducted a *t* test to assess whether the mean difference was different from zero. Again, we did this to ensure that beauty ratings were not significantly higher or lower in a specific block. We also conducted an *F* test to assess the equivalence of the variances of those distributions (which assess whether the true ratio of variances is equal to 1). Such test assessed whether order contributed significantly to the variance of the ratings.

A concern with this approach is the possibility that contrast and assimilation effects nicely cancel each other out, resulting in no difference in variability. Hence, to directly assess sequential dependence, we measured the extent to which the rating of the preceding image predicted the rating of each image. We did this for the scrambled block using a linear mixed-effect model. We included participants and images as random intercepts. We included the initial beauty rating of the target image and the preceding image’s initial (first block) and recent ratings (scrambled block) as fixed factors. Since the images in the unscrambled block are in the same order as in the initial block, then we cannot separate the responses from the stimulus. Thus, we only conducted the analysis using the ratings from the scrambled block.

### Results

**Fig. 4:**
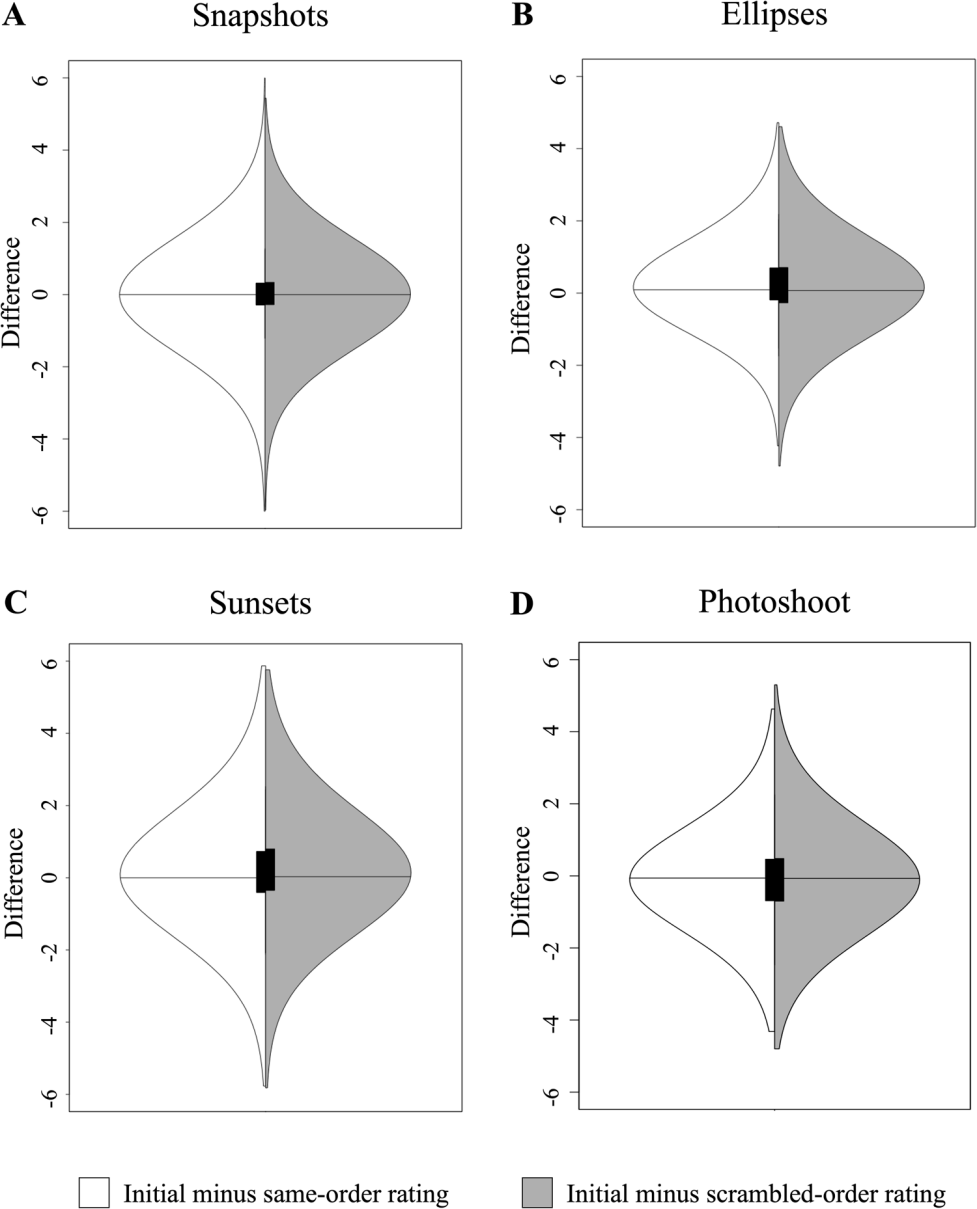
Violin plots of the differences between the initial and scrambled-order (white) and same-order (gray) ratings for beauty of OASIS images (**A**; Experiment 3), ellipticity (**B**; Experiment 4), beauty of sunsets (**C**; Experiment 5), and beauty of photoshoot images (**D**; Experiment 6). The horizontal lines correspond to the medians and the vertical black rectangles correspond to the interquartile range

We did not find a difference between the variance of the difference in beauty ratings in the same-order and scrambled blocks, *F*(3749, 3749) = 0.945, *p* = 0.085, 95% CI [0.887, 1.008]. For the same-order block, the standard deviation of the difference was 0.87, and for the scrambled block, the standard deviation of the difference was 0.90. Moreover, the mean difference was not significantly different from zero for both the same-order block, *t*(3749) = −0.768, *p* = 0.443, 95% CI [−0.039, 0.017], and the scrambled block, *t*(3749) = 1.278, *p* = 0.201, 95% CI [−0.010, 0.048]. Figure 4A displays the distributions of difference for both the scrambled and same-order blocks.

Previous research has shown that contrast effects result from the perceptual adaptation of the stimulus, while assimilation effects result from the motor repetition of the response (Huang et al., 2018). Thus, we assessed sequential dependence by how well the rating in the scrambled block was predicted by the preceding image’s *recent* and *initial* ratings. Since the preceding image’s recent rating was the immediately previous response, correlation with this rating indicates an effect of that response. In contrast, correlation with the preceding image’s initial rating indicates an effect of that stimulus’ perception.

A contrast effect (negative dependence) would indicate that participants tend to bias their responses away from their preceding rating (e.g., a beautiful image is more highly rated after a plain image). An assimilation effect (positive dependence) would indicate that participants tend to bias their responses towards their preceding rating (e.g., a beautiful image is rated slightly less beautiful after a plain image).

We found no significant dependence of the current-image rating on either the new or initial rating of the previous image. Though it is expected that new and initial ratings of the previous image are strongly correlated, their *variance inflation factors* (*VIF*) are 4.87. The VIF for a regressor is the variance of the estimate that uses all the regressors divided by the variance of the estimate using just that regressor. The VIF measures collinearity. A VIF below 10 indicates that the variables are independent and thus can be used in a linear model without fear of undue bias (Hair et al., 1992). Table 7 shows the details of the model.

**Table 7:** Mixed-effects model for sequential dependence in beauty ratings

Random Effects (Intercepts)					
	Variance	*SD*			
Image	0.04	0.2			
Participant	0.04	0.2			
Fixed Effects					
	Estimate	*SE*	*df*	*t*	*p*
**Intercept**	**0.37**	**0.06**	**245.1**	**6.48**	**<0.001**
**Initial Beauty Rating**	**0.86**	**0.01**	**940.5**	**95.41**	**<0.001**
Rating of Previous Image	0.02	0.02	3649	1.08	0.28
Initial Rating of Previous Image	0.02	0.02	3648	1.10	0.27

The model explains 82.6% of the variance in beauty ratings with an RMSE of 0.829

Bold indicates significance. The new and initial beauty ratings of the previous image had no significant contrast or assimilation effect on the beauty rating of the current image

### Discussion

The test–retest variability of beauty ratings was the same whether the image order was conserved or scrambled. Thus, sequential dependence does not significantly influence the variance of repeated beauty ratings. Moreover, our mixed-effects linear model indicates that our results are not the result of opposite contrast and assimilation effects canceling each other, because our linear model found neither contrast nor assimilation effects.

#### Similarity hypothesis

These results are unexpected and go against previously observed assimilation and contrast effects in beauty judgments (e.g., Huang et al., 2018; Kondo et al., 2012; Kramer & Pustelnik, 2021; Tousignant & Bodner, 2014). However, their experiments used stimuli that were very similar to each other (e.g., faces). Researchers have reported that the similarity between the stimuli modulates these order effects (Damisch et al., 2006; Dolese et al., 2005). The images in our experiment represent different object categories and do not invite comparison. Hence, we hypothesized that stimulus similarity influences the magnitude of sequential effects. Specifically, if we were to conduct the same experiment using stimuli with high similarity, we would observe significant contrast effects for the initial rating of the previous image and assimilation effects for the rating of the previous image.

To test our hypothesis, we conducted the same experiment on the ellipse stimuli used in Experiment 2. These vary only on aspect ratio and color, and hence have high similarity.

## Experiment 4: Sequential dependence in ellipticity ratings

### Methods

#### Participants

Fifty new participants took part in the experiment. Twenty-one identified themselves as female, 26 as male, one as other, and two decided not to say. Their ages ranged from 18 to 65 years (*M* = 29.5, *SD* = 9.54). All participant selection and recruitment were the same as in the experiments above.

#### Stimulus and apparatus

The stimuli for this experiment were the exact same images as in Experiment 2. Experiment 4 was programmed and distributed in the same way as all previous experiments.

#### Procedure

The experimental design was equal to that of Experiment 3, except that participants rated ellipticity instead of beauty. Thus, to give participants an anchoring of our scale, we added a training block at the beginning of the survey that resembled the training block in Experiment 2.

### Results

We excluded the trials of the participants who did not answer the comprehension-check trials correctly (15 participants, new *N* = 35). We found a significant difference in the variabilities of the difference in ellipticity rating in the same-order and scrambled blocks, *F*(2624, 2624) = 0.798, *p* < 0.001, 95% CI [0.740, 0.862]. For the same-order block, the standard deviation of the difference was 0.92, and for the scrambled block the standard deviation of the difference was 1.03. Moreover, the mean difference significantly differed from zero for both the same-order block, *t*(2624) = 13.337, *p* < 0.001, 95% CI [0.204, 0.275], and the scrambled block, *t*(2624) = 8.968, *p* < 0.001, 95% CI [0.141, 0.220]. This means that on average, all else being equal, participants tended to give lower ellipticity ratings to ellipses in the second and third blocks. Figure 4B displays the distribution of differences for both the scrambled and same-order blocks. Using the same criteria as in Experiment 3 (see also Huang et al., 2018), the results of our mixed-effect model indicate significant contrast and assimilation effects. Table 8 shows the details of the model. On average, the initial ellipticity rating and the rating of the previous image had a significant positive effect on the ellipticity rating in the scrambled block. Furthermore, on average, the initial rating of the previous image had a significantly negative effect on the ellipticity rating in the scrambled block. Hence, we observe an assimilation effect stemming from anchoring to the previous rating and a contrast effect stemming from perceptual adaptation to the previous stimulus. Once again, the VIF for the rating of the previous image and the initial rating of the previous image, 3.413 for both, are acceptable (Hair et al., 1992)**.**

**Table 8:** Mixed-effects model for sequential dependence in ellipticity ratings

Random Effects (Intercepts)					
	Variance	*SD*			
Image	1.232	1.12			
Participant	0.198	0.69			
Fixed Effects					
	Estimate	*SE*	*df*	*t*	*p*
Intercept	**2.48**	**0.17**	**157.9**	**14.26**	**<0.001**
Initial Ellipticity Rating	**0.28**	**0.02**	**2442**	**15.16**	**<0.001**
Rating of Previous Image	**0.22**	**0.02**	**2493.8**	**14.15**	**<0.001**
Initial Rating of Previous Image	**-0.09**	**0.01**	**2484.6**	**-6.33**	**<0.001**

The model explains 79.6% of the variance in ellipticity ratings with an RMSE of 0.672

Bold indicates significance. We found a significant assimilation effect of the rating of the previous image and a significant contrast effect of the initial rating of the previous image

### Discussion

We observed differences between ellipticity and beauty ratings in terms of the sequential dependence and how these affect measures of intrinsic variance. For beauty ratings, we found the same variability of beauty ratings in the same-order versus the scrambled-order blocks, but for ellipticity ratings, order mattered. These results hint that the influence of sequential dependence on the intrinsic variability of beauty may depend on stimulus similarity. However, the difference between the two tasks is a possible confound with our hypothesis that sequential dependence depends on stimulus similarity. In Experiment 3 we asked participants to rate beauty, but in Experiment 4 we asked participants to rate ellipticity. Moreover, similarity between stimuli may be conceptualized in various ways. On one hand, there can be semantically similar images. This refers to stimuli that convey the same object category. On the other hand, there can be similar images of the same subject. For example, there could be changes in the lighting on the orientation of the photo. In order to address this confound and test our hypothesis that the presence of sequential dependence depends on stimulus similarity, we conducted Experiments 5 and 6. In Experiment 5, we asked participants to rate the beauty of images of sunsets (all images conveyed the same object category). In Experiment 6, we asked participants to rate the beauty of images from a fashion photoshoot (all images were of the same subject).

## Experiment 5: Sequential dependence in beauty ratings of semantically similar stimuli

### Methods

#### Participants

Fifty-one new participants took part in the experiment. Thirty-five identified themselves as female and 16 as male. Their ages ranged from 18 to 70 years (*M* = 34.45, *SD* = 11.52). All participant selection and recruitment were the same as in the experiments above.

#### Stimulus and apparatus

We obtained 75 images of sunsets from Unsplash (https://unsplash.com/), a website of free, openly usable stock photos. All photos have a landscape orientation and display a sunset (including the sun) as the main focal element of the photo. The links to the photos and the names of the photographers can be found here: https://osf.io/wecvp/.

Experiment 5 was programmed and distributed in the same way as all previous experiments.

#### Procedure

The experimental design was equal to that of Experiment 3.

### Results

We did not find a difference in the variability of the difference in beauty rating in the same-order and scrambled blocks, *F*(3824, 3824) = 1.00, *p* = 0.9288, 95% CI [0.94, 1.07]. For the same-order block, the standard deviation of the difference was 1.312, and for the scrambled block, the standard deviation of the difference was 1.310. Moreover, the mean difference was significantly different from zero for both the same-order block, *t*(3824) = 8.22, *p* < 0.001, 95% CI [0.13, 0.22], and the scrambled block, *t*(3824) = 10.64, *p* < 0.001, 95% CI [0.18, 0.27]. This is indicative that on average, all else being equal, participants gave lower beauty ratings on the second and third blocks. Figure 4C displays the distributions of difference for both the scrambled and same-order blocks. Using the same criteria as in Experiments 3 and 4 (see also Huang et al., 2018), the results of our mixed-effects model indicate significant assimilation effects. However, the results do not indicate a significant contrast effect. Table 9 shows the details of the model. On average, the initial beauty rating and the rating of the previous image had a significant positive effect on the beauty rating in the scrambled block. Furthermore, the initial rating of the previous image did not have an effect on the beauty rating in the scrambled block. Once again, the VIF for the rating of the previous image and the initial rating of the previous image, 1.57 for both, are acceptable (Hair et al., 1992).

**Table 9:** Mixed-effects model for sequential dependence in beauty ratings of similar stimuli

Random Effects (Intercepts)					
	Variance	*SD*			
Image	0.09	0.29			
Participant	0.21	0.46			
Fixed Effects					
	Estimate	*SE*	*df*	*t*	*p*
Intercept	**1.38**	**0.12**	**336.32**	**11.01**	**<0.001**
Initial Beauty Rating	**0.59**	**0.01**	**3401.67**	**42.13**	**<0.001**
Rating of Previous Image	**0.12**	**0.02**	**3734.42**	**7.30**	**<0.001**
Initial Rating of Previous Image	−0.02	0.02	3743.14	−0.963	0.335

The model explains 54.8% of the variance in beauty ratings with an RMSE of 1.087

Bold indicates significance. The results suggest a significant assimilation effect of the rating of the previous image but so significant contrast effects

### Discussion

Consistent with our similarity hypothesis, we observed assimilation effects of the previous rating on beauty ratings of semantically similar images. However, we did not observe a significant contrast effect of the initial rating of the previous image on the beauty rating of semantically similar images. Notably, the difference between the variability of the distribution of difference was not significant, indicating that even when the stimuli were semantically similar, the observed assimilation effect has a negligible effect on the variability of repeated measures.

## Experiment 6: Sequential dependence in beauty ratings of similar images of the same subject

### Methods

#### Participants

Fifty new participants took part in the experiment. Twenty-four identified themselves as female, 23 as male, one as other, and one preferred not to say. Their ages ranged from 19 to 62 years (*M* = 35.48, *SD* = 12.39). All participant selection and recruitment were the same as in the experiments above.

#### Stimulus and apparatus

We obtained 75 images from a fashion photoshoot. All the pictures were of the same model, wearing the same outfit, changing poses slightly between every picture. All photos have a portrait orientation, and the model is always posing and standing in the center. The lighting was held constant. The links to the photos and the names of the photographer and model can be found here: https://osf.io/wecvp/.

#### Procedure

The experimental design was equal to that of Experiments 3 and 5.

### Results

We excluded the data from one participant who gave the same rating to all images in all blocks and therefore was not following instructions (thus, *N* = 49). We found a significant difference in the variability of the differences in beauty rating in the unscrambled and scrambled blocks, *F*(3674, 3674) = 0.89, *p* < 0.001, 95% CI [0.83, 0.95]. For the unscrambled block, the standard deviation of the differences was 1.13, and for the scrambled block, the standard deviation of the differences was 1.20. Moreover, the mean difference was significantly different from zero for both the unscrambled block. *t*(3674) = −5.40, *p* < 0.001, 95% CI [−0.14, −0.06], and the scrambled block, *t*(3674) = −3.95, *p* < 0.001, 95% CI [−0.12, −0.04]. This means that, on average, participants reported higher ratings during the second and third blocks. Figure 4D displays the distributions of difference for both the scrambled and same-order blocks. Using the same criteria as in Experiments 3–5 (see also Huang et al., 2018), the results of our mixed-effects model indicate significant contrast and assimilation effects. Table 10 shows the details of the model. On average, the initial beauty rating and the rating of the previous image had a significant positive effect on the beauty rating in the scrambled block. Furthermore, the initial rating of the previous image had a significantly negative effect on the beauty rating in the scrambled block. Hence, we observe an assimilation effect stemming from anchoring of the previous judgment and a contrast effect stemming from the perceptual properties of the previous stimulus. Lastly, the VIF for the rating of the previous image and the initial rating of the previous image, 1.33 for both, are acceptable (Hair et al., 1992).

**Table 10:** Mixed-effects model for sequential dependence in beauty ratings of similar stimuli

Random Effects (Intercepts)					
	Variance	*SD*			
Image	0.06	0.24			
Participant	0.28	0.53			
Fixed Effects					
	Estimate	*SE*	*df*	*t*	*p*
Intercept	**1.76**	**0.12**	**197.07**	**14.28**	***p*** **<0.001**
Initial Beauty Rating	**0.45**	**0.01**	**3334.48**	**31.84**	***p*** **<0.001**
Rating of Previous Image	**0.18**	**0.02**	**3582.92**	**11.55**	***p*** **<0.001**
Initial Rating of Previous Image	−**0.03**	**0.02**	**3589.68**	−**1.97**	**0.049**

The model explains 55.6% of the variance in beauty ratings with an RMSE of 0.927

Bold indicates significance. The results suggest a significant assimilation effect of the rating of the previous image and a significant contrast effect of the initial rating of the previous image

### Discussion

Consistent with our similarity hypothesis, for beauty ratings of similar images of the same subject, we observed an assimilation effect of the new rating of the preceding image and a contrast effect of its initial rating. In particular, for similar stimuli, scrambling the order significantly increased the variance of the test-retest difference. Thus, the results of Experiments 5 and 6 suggest that physical similarity between stimuli, not just semantic similarity, significantly affects the variance of repeated measures.

**Fig. 5:**
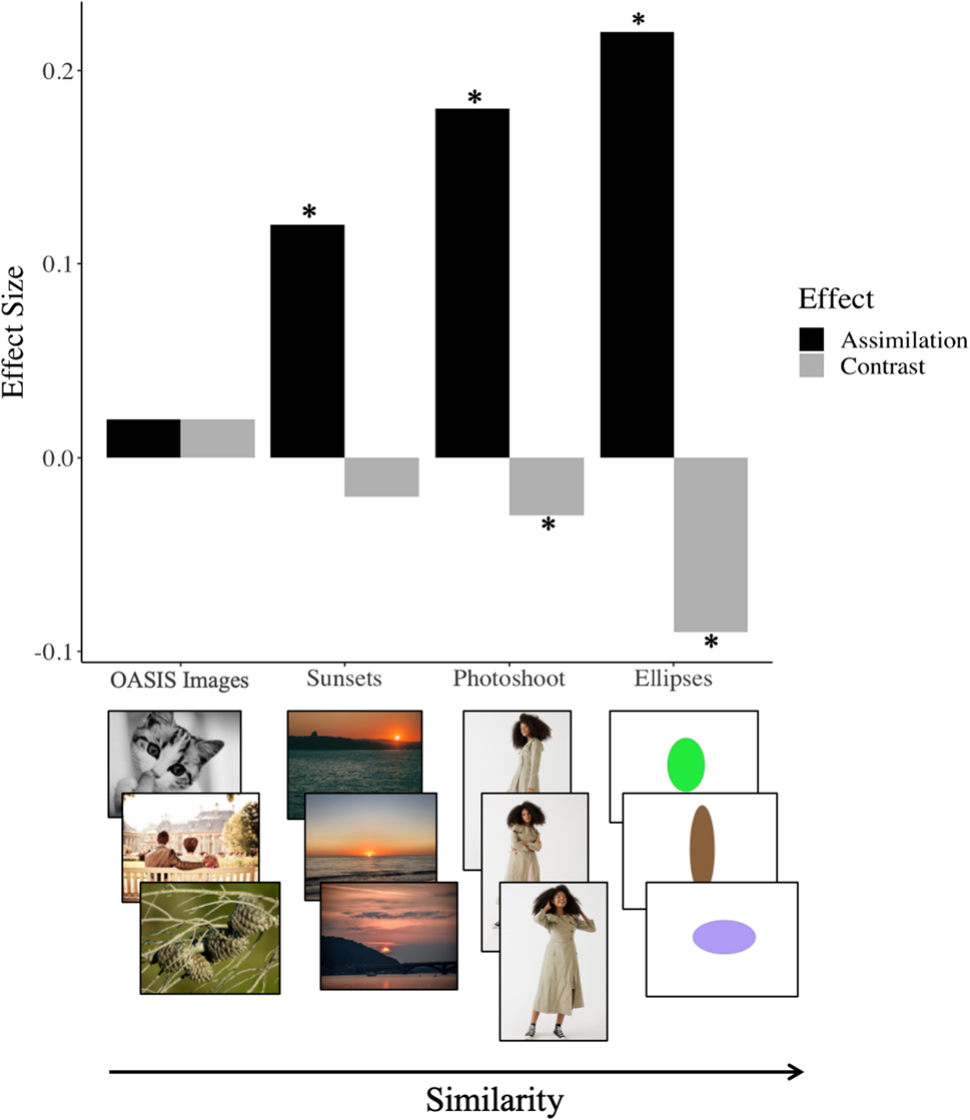
Contrast and assimilation effect as a function of similarity. The *x*-axis represents Experiments 2, 5, 6, and 3, from left to right. As similarity between stimuli increases, the magnitude of both assimilation and contrast effects increases, regardless of task. * indicates *p* < 0.05. Similarity has a significant effect as indicated by the star, independently for each of the four image kinds. (Color figure online)

The magnitudes of the assimilation and contrast effects are small, but the results of Experiments 3–6 indicate that they become larger as stimuli become more similar, regardless of task (see Fig. 5).

## Experiment 7: Intrinsic variance as a function of stimulus set size

Since the number of images used in our experiments was somewhat arbitrary, we chose to assess the generality of our findings by measuring the effect of recall memory over a range of stimulus set sizes. Having more stimuli increases interference by other stimuli and thus may influence the effect of recall memory on variability.

### Methods

#### Participants

We recruited an additional 50 participants for this experiment. Twenty-four of them identified themselves as female, and 26 as males. Their ages ranged from 19 to 70 years (*M* = 35.2, *SD* = 13.63). All participant selection and recruitment were the same as in the experiments above.

#### Stimuli and apparatus

The stimuli for this experiment were the same set of images as in Experiment 1. They were programmed and distributed in the same way as in Experiment 1.

#### Procedure

The procedure was the same as in Experiment 1. All participants completed the same four blocks (initial rating, repeat, memory, and memory check). This experiment differed in the number of images in each block. Participants were randomly assigned to two groups: Half of the participants completed the experiment with a set size of 9 images and the other half completed the experiment with a set size of 1 image. With these stimulus set sizes, we measure stimulus set size along a log scale between 1 and 75. For the experiment to last a similar amount of time as Experiment 1 and for participants to rate the same number of images in total, participants in the one-image condition completed the four blocks, 75 times, and participants in the nine-image condition completed the four blocks a total of eight times (the last set of blocks had an image set size of 12).

#### Analysis

We were interested in looking at the variability of beauty ratings as a function of set size for both the repeat block and the memory block. To assess the variability, we calculated the distribution of difference between the initial beauty rating and the beauty rating of the repeat or the memory blocks in the same way as in Experiment 1. Moreover, we assessed the standard deviation of these distributions as a function of set size. Lastly, we calculated the variance of the immediate beauty perception as a function of set size.

### Results

**Fig. 6:**
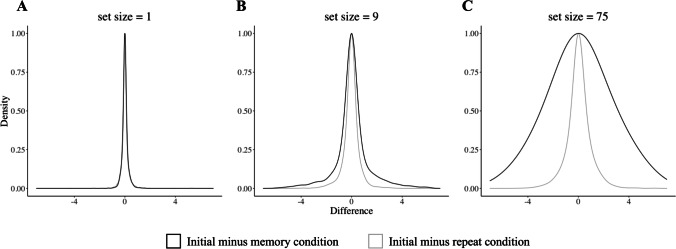
Density distributions of the difference as a function of set size. The gray distributions correspond to the repeat blocks and the black distributions correspond to the memory blocks. The first column corresponds to a stimulus set size of 1 image, the middle column corresponds to a set size of 9 images, and the third column corresponds to a set size of 75 (Experiment 1, see Fig. 2)

We only considered the trials for which participants remembered which image was associated with each name correctly. For the one-image condition, this constituted 99.9% of the data (99.8% when corrected for guessing). For the nine-image condition, this constituted 91.4% of the data (87.1% when corrected for guessing). For the one-image condition, the standard deviation of the difference of the repeat block (initial rating − repeat rating) is 0.24, while for the memory block (initial rating − memory) the standard deviation is 0.28. The difference between the two is significant, *F*(1874, 1874) = 0.780 , *p* < 0.001, 95% CI [0.712, 0.854]. For the nine-image condition, the standard deviation of the repeat block is 0.64, and 1.37 for the memory block. Once again, the difference between the two is significant, *F*(1713, 1713) = 0.219, *p* < 0.001, 95% CI [0.200, 0.241]. Figure 6 shows the density distributions of the difference as a function of stimulus set size.

**Fig. 7:**
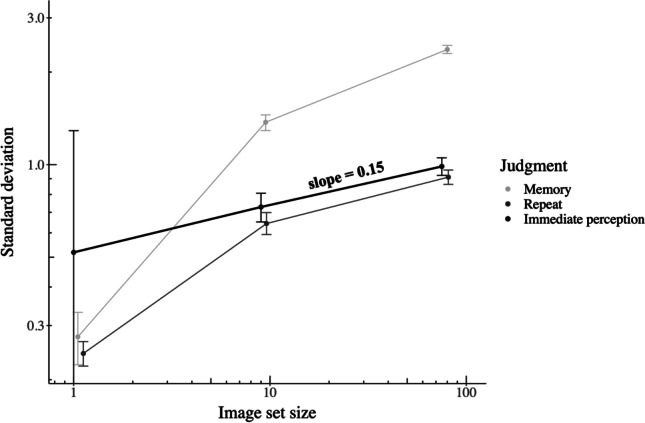
Standard deviations of the difference for the memory block, repeat block, and for the immediate-perception judgment as a function of stimulus set size, plotted on log-log scale. Error bars represent 95% confidence intervals of 500 bootstrap samples. The plot indicates an increase in repeated measures variance even after discounting the effect of memory

For each set size, we calculated the standard deviation of the immediate-perception judgment (*σ*_*I*_). For a set size of 1 image, *σ*_*I*_ is 0.52 (0.28 increase from *σ*_*R*_). For a set size of nine images, *σ*_*I*_ is 0.73 (0.09 increase from *σ*_*R*_). For a set size of 75 images, *σ*_*I*_ is 0.99 (see Experiment 1). Figure 7 shows the standard deviations of the repeat and memory blocks, as well as the calculated standard deviation of the immediate-perception judgment, plotted as a function of set size on a log-log scale. The line of best fit of the standard deviation of the immediate perception judgments has a slope of 0.15. In a log-log scale, the slope of the line corresponds to the power of the relationship, so standard deviation increases in proportion to the 0.15 power of stimulus set size.

### Discussion

In our results, increasing set size increased both the variance of repeated beauty rating and the variance of the difference between a beauty rating and a rating relying only on recall memory. After using Bayesian optimal cue combination to discount the effect of memory (Equation 1), the intrinsic standard deviation of beauty ratings increases as the 0.15 power of stimulus set size. It is worth noting that with our design, the time between measures is proportional to stimulus set size. The variance of repeated measures may increase when there is additional time between measures, even when there are no additional stimuli. Further research is needed to tease apart the effects of stimulus set size versus time between measures on the variance of repeated measures. For example, adding a distraction task between measures could allow manipulating stimulus set size while keeping time between measures constant.

## General discussion

This study estimates the intrinsic variance of a beauty rating, which is estimated by the variance of repeated measures only if the measures are independent. So, we assessed the independence of repeated measures. We measured the effects on variance of both memory and sequential dependence, two potential threats to independence. Our results indicate stimulus similarity and set size modulate the effect of order effects, including memory, on the variance of the test–retest difference. Thus, testing 75 diverse stimuli, we found that the variance of repeated measures of beauty appropriately estimates the intrinsic variance of a beauty rating.

### Memory

We found that with a set size of 1, memory is a problem and contributes half the measured variance. However, for both beauty and ellipticity judgments with set size of at least 9, recall memory makes only a small contribution to repeated judgment. In our case, recall memory contributed less than 10% of the variance. Even though there may be cases where a fractional contribution may have practical implications (e.g., in sales), to us, 10% of the variance is negligible because aestheticians hardly ever measure anything with such precision. Moreover, we showed that regardless of the objectivity of a judgment (and availability of ground truth), one can estimate how much recall memory reduces the variance of repeated judgments by using a paired-association cue recall task. That contribution may then be discounted using the Bayesian cue-combination rule to estimate the immediate-perception variance (Alais & Burr, 2019; Ernst & Banks, 2002; Oruç et al., 2003). Our method improves on previous attempts to calculate the effect of recall memory on repeated measures (Schwarz et al., 2020; van Meurs & Saris, 1995) by including repeated measures for multiple stimuli within a single experiment and taking into account the variance of the measurements.

Despite all our efforts, one can still wonder what participants remember. Concerned that participants might remember the image and forget the name label, we base our conclusions on the substantial fraction (60% for images and 39% for ellipses) of trials for which they did remember the label. More generally, our conclusions rest on our results and two assumptions: (1) The reliabilities of the memory of the image (and rating) and perception of the image are additive. (2) Restricting the sample to cases in which, having seen several named images, participants can identify which image is associated with any given name, the variance in rating is primarily limited by the faithfulness of the image (and rating) memory and not the name memory, especially after correcting for guessing. The validity of our conclusions requires only our results and the validity of those two assumptions.

### Similarity

Previous reports of order effects on beauty judgments used very similar stimuli (Huang et al., 2018; Tousignant & Bodner, 2014), and stimulus similarity modulates order effects (Damisch et al., 2006; Dolese et al., 2005). The results of Experiment 3 indicate no order effects on beauty judgments of varied images and no influence of order effects on the variability of repeated measures of beauty judgment. Experiment 4 found significant order effects on ellipticity rating, which significantly affect the variance of repeated ratings. When only considering semantic similarity (Experiment 5), we observed a significant assimilation effect but no significant contrast effect. However, the results of Experiment 5 indicate that the effect negligibly affects the variance of repeated beauty ratings (0.15%). When considering similar images of the same subject (Experiment 6), we observed order effects on beauty judgment. These order effects significantly affected the variance of repeated beauty ratings. Overall, the results of Experiments 3–5 suggest that the magnitude of assimilation and contrast effects increase with stimuli similarity. Moreover, our results are consistent with previous accounts that propose that the influence of order effects on the variability of judgments depends on the similarity of stimuli.

### Set-size dependence

We found that the beauty judgment of a stimulus is affected by stimulus diversity and set size, and unaffected by order of presentation. Our results indicate that the response to a given stimulus in a diverse set is affected by the *number* and not the *value* of other stimuli. If the response were affected by the value of the other stimuli, order of stimuli presentation would affect the variance of repeated measures of beauty judgment. However, when the stimuli are similar, values do matter. The fact that the standard deviation increases with set size suggests that some resource (e.g., attention) is spread more thinly across stimuli when there are more stimuli.

### Modeling aesthetic value

An alternative explanation for the set-size effect, as well as the selective presence of sequential dependence in homogeneous stimulus sets, could be found in a recent theory of aesthetic value (Brielmann & Dayan, 2021). The theory is based on the idea that stimuli are encoded in terms of the probability of their features, and that the underlying joint probability distribution constitutes the observer’s internal model of the sensory world. Aesthetic value, and hence beauty, is proportional to the object’s likelihood given the model. Crucially, the model is updated based on the observer’s experience (i.e., experienced features become more probable, the more so, the longer the experience). Due to its learning component, the theory predicts more systematic changes in beauty judgments when stimuli are homogeneous (if stimuli vary along the same features, learning always affects these features and hence changes ratings systematically) and when more stimuli have been presented in between ratings (more exposure leads to more learning). Since the feature space of all the images used in our beauty judgment experiments is potentially vast and ill-defined (i.e., even in the photoshoot images the images vary in infinite ways), we cannot fit this model to the dataset presented here. Future experiments with specifically designed stimulus material might determine whether the model can quantitatively predict the effects we observed here.

## Conclusion

Recall memory and sequential dependence threaten the independence of successive beauty ratings. Such independence is usually assumed when using repeated measures to estimate the intrinsic variance of a rating. With a set size of 1, memory is a problem and contributes half the measured variance. However, we showed that for both beauty and ellipticity, with set size of 9 or more, recall memory causes a mere 10% increase in the variance of repeated ratings. Moreover, we showed that as long as the stimuli are diverse (i.e., represent different object categories), sequential dependence does not affect the variance of beauty rating. Lastly, this variance increases in proportion to the 0.15 power of stimulus set size. We show that the beauty rating of a stimulus in a diverse set is affected by the stimulus set size and not the value of other stimuli. Overall, we conclude that the variance of repeated ratings is a good way to estimate the intrinsic variance of a beauty rating of a stimulus in a diverse set.
